# Characterisation and FE Modelling of the Sorption and Swelling Behaviour of Polyamide 6 in Water

**DOI:** 10.3390/polym13091480

**Published:** 2021-05-04

**Authors:** Anna Katharina Sambale, Michael Stanko, Jessica Emde, Markus Stommel

**Affiliations:** 1 Institute of Polymer Materials, Leibniz-Institut für Polymerforschung Dresden e.V., Hohe Str. 6, D-01069 Dresden, Germany; stommel@ipfdd.de; 2 Chair of Plastics Technology, TU Dortmund University, Leonard-Euler Str. 5, D-44227 Dortmund, Germany; michael.stanko@tu-dortmund.de (M.S.); jessica.emde@tu-dortmund.de (J.E.)

**Keywords:** polyamide 6, water sorption, moisture-induced swelling, concentration-dependent diffusion coefficients, FE modelling

## Abstract

Polyamide 6 (PA6) is known to absorb water from its environment due to its chemical structure. This water absorption leads to a change in the mechanical properties as well as an increase in volume (swelling) of the polyamide. In the present work, the sorption and swelling behaviour of polyamide 6 in different conditioning environments was experimentally investigated on different part geometries to develop a finite element (FE) method on the basis of the measured data that numerically calculates the sorption and swelling behaviour. The developed method includes two analyses using the Abaqus software. Both the concentration-dependent implementation of the simulation parameters and the calculation of swelling-induced stresses are performed. This enables the modelling of the sorption curves until maximum saturation is reached and the simulation of the characteristic S-shaped swelling curves. Therefore, the developed methodology represents an efficient method for predicting the sorption and swelling behaviour of polyamide 6 parts during conditioning in a water bath. The determined properties provide the basis for the development of an FE-based simulation environment to take moisture absorption into account during the part design. This enables the calculation of moisture-induced swelling processes and the resulting initial stresses in a given part.

## 1. Introduction

Polyamide 6 (PA6) is able to absorb moisture from the surrounding air and release it again until it is in balance with the environment. This effect is based on the diffusion processes of polar water molecules, which are attracted by the similarly polar amide groups of the molecule chains [1]. The water molecules can be bound by hydrogen bonds between the amides and increase the chain segment mobility due to the increased molecular chain spacing [2,3]. This lowers the glass transition temperature of the material from about 60 °C for dry PA6 to about −20 °C for completely water-saturated material [4,5,6,7,8,9]. This is referred to as the water-induced plasticising effect, which significantly reduces the stiffness and strength of PA6 and significantly increases the (fracture) toughness [10,11,12,13]. The diffusion process of the water into the bulk material is influenced by various parameters, such as temperature, ambient water concentration, load, and diffusion path of the water, until the concentration is balanced [14,15,16,17]. At room temperature and low water concentration in the air, it can take years until the material is in equilibrium with the environment [18,19]. At high water concentrations inside the material and low ambient (air) humidity, the water is also released back into the environment by diffusion-controlled desorption processes. The water absorption of PA6 is therefore a reversible process: until the concentration of the material is in equilibrium with the environment, there are moisture gradients that also cause gradients in the material properties due to the water-induced plasticisation [17,20,21,22].

Due to the additional space required for depositing the water molecules in the amorphous area of the semicrystalline PA6, moisture absorption also causes an increase in volume (swelling) of the material, which also has a significant effect on the dimensional accuracy of the PA6 parts and causes internal stresses [23]. However, to be technically applicable and to prevent continuous changes in part dimensions and properties during use, dry PA6 is usually conditioned before use (ISO 20457). For this purpose, there are various methods, each based on storing the material at elevated temperatures and humidity levels until the weight of the part is constant, or, optionally, for a defined period of time in a heated water bath until a desired integral water content is achieved [18]. During conditioning, the aim is mostly to reach a state of equilibrium with a standard climate of 23 °C and 50% relative humidity (r.h.), resulting in water-induced weight increases of about 2.5–3% [18,24]). To control the state of conditioning and to reduce the humidity gradients, all conditioning methods suggest storage in a humidity chamber controlled to a standard climate while weighing the conditioned parts at defined intervals. If the weight of the part does not change further over a certain period of time, the material is in equilibrium with its environment [19]. Nevertheless, after the conditioning and storage process, moisture gradients may exist or uncontrolled overconditioning may occur, which makes it difficult to precisely predict the change in properties and dimensions of parts in environments exposed to moisture [12]. A prediction of the water sorption and the resulting swelling behaviour can help to optimise the design PA6 parts.

During the dimensioning of plastic parts, different environmental influences that change the properties of a part in application have to be taken into account. The consideration of process- and material-specific part properties, such as shrinkage-induced residual stresses or the fibre orientation of a fibre-reinforced plastic part, are extensively established in computer-aided part design in the engineering practice. Other factors, such as the application of temperature or UV exposure, can also be included in current FE analyses through corresponding model-based changes in the mechanical properties of a plastic part. In the case of PA6, the most significant environmental factor is the influence of moisture. It should be noted that there is a significant amount of research [14,16,23,25] concerning the computer-aided calculation of the water absorption of plastic parts, but that these are often limited to simplified test geometries. The general objective in this field of research is to develop a computer-aided calculation method for accurately mapping the water absorption of plastic parts with complex geometries (as are typically found in real-world applications). Due to the design complexity of plastic parts with varying wall thicknesses and part contours, the determination of the moisture absorption and the resulting changes in the part dimensions constitutes a particular challenge.

Taking these influences into account through classical approaches, such as adding suitable tolerances to the components’ geometry (e.g., to take into account the manufacturing shrinkage), is considered to be of very limited practicality. Furthermore, the corresponding standards, e.g., for the tolerances of plastic parts in ISO 20457, do not refer to the influence of moisture on the application-specific part properties. Within the scope of this contribution, experimental basics for the development of a computer-aided FE calculation methodology are presented using the example of a step-shaped part with different wall thicknesses.

## 2. Calculation Basics on the Sorption and Swelling Processes

### 2.1. Calculation Basics on the Sorption Process

It is known from the literature that the water absorption process of PA6 shows Fick’s diffusion behaviour in the initial region of the sorption curve [26]. This is generally described with the aid of two laws, where Fick’s 1st law (Equation (1)) describes the stationary diffusion of liquids, gases, and vapours in a homogeneous continuum and Fick’s 2nd law (Equation (2)) refers to time-dependent diffusion.

(1) J=−D·∇c

(2) $$\frac{\partial c}{\partial t}=\nabla \left(D\cdot \nabla c\right)$$

Here, J [m/s] is the concentration flow, which defines the volumetric flow of particles over an area, $D\;[m^{2}/s$] describes the diffusion coefficient, and c [%] the concentration of the diffusing substance in the solid. Since only the sorption of water in polyamide 6 is considered here, a single diffusing substance can be assumed. In addition, it is initially assumed that the diffusion coefficient is independent of the concentration and only one-dimensional diffusion in the x-direction is considered. Under the assumption of isotropic diffusion properties in the solid (∇D=0) and a constant concentration of the diffusing substance in the considered period of diffusion (dc/dt=0), Fick’s laws can be further simplified, according to Crank [27,28] and Menges [26]:

(3) $$J=-D\;\frac{\partial c}{\partial x}$$

(4) $$\frac{\partial c}{\partial t}=D\;\frac{\partial^{2}c}{\partial^{2}x}$$

Using various boundary conditions, the Fick’s 2nd law can be solved analytically by applying a Fourier series:
(1)$c\left(x,t\right)=c_{0}=0$for $-\frac{d}{2}<x<\frac{d}{2}$
at t=0
(2)$c\left(x,t\right)=c_{\infty }$for $x=\frac{d}{2}\;\;\cap \;x=-\frac{d}{2}$
at $0<t<t_{\infty }$
(3)$c\left(x,t\right)=c_{\infty }$for $-\frac{d}{2}<x<\frac{d}{2}$
at $t=t_{\infty }$



**Figure 1:**
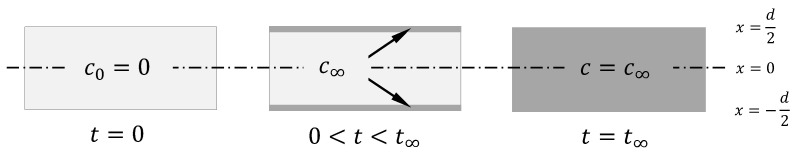
Schematic diagram of the sorption process for different exposure times.

At the start of diffusion (t=0), a sample of thickness d [m] has a constant concentration of $c_{0}=0$ (1), as shown in Figure 1. It is then exposed to an environment with the concentration $c_{\infty }$ of the diffusing substance so that for each subsequent point in time the concentration at the surface of the sample corresponds to the concentration of the surrounding medium (2). After a period of time $t_{\infty }$, where no significant changes in the material properties can be detected, a state of equilibrium is reached where the saturation concentration for the entire sample is $c_{\infty }$ (3) [27,28].

The solution of this system of equations can be found in detail in the study by Crank [27,28] and is dependent on the thickness d [m] of the test specimen:

(5) $$\frac{c\left(x,\;t\right)}{c_{\infty }}=1-\frac{4}{\pi }\;\overset{\infty }{\underset{n=0}{\sum }}\frac{{\left(-1\right)}^{n}}{2n+1}\cdot \exp\left(\frac{-D\;{\left(2n+1\right)}^{2}\;\pi^{2}\;t}{d^{2}}\right)\cdot \cos\left(\frac{\left(2n+1\right)\;\pi \;x}{d}\right)$$

The water sorption process can be verified experimentally by recording the time-dependent increase in the weight of a sample. It is typical to relate the percentage mass change $M_{t}\;\left[\%\right]$ at the current time t to the maximum mass change $M_{max}\;\left[\%\right]$ in the saturated state. $M_{t}$ is calculated as follows:

(6) $$M_{t}=\frac{m_{t}-m_{0}}{m_{0}}\cdot 100$$

with the mass $m_{0}\;\left[\mathrm{kg}\right]$ of the dry specimen and the mass $m_{t}$ [kg] at the considered time t [15]. According to Vlasveld et al. [14], the maximum mass change $M_{max}$ in the saturated state can also be represented by the product of the percentage change in density per unit volume Δρ [$\%/m^{3}$] and the volume of the test specimen $V\;[m^{3}$]:

(7) $$M_{max}=\Delta \rho \cdot V$$

For the initial phase of sorption up to $M_{t}/M_{max}=0.5$, the following relationship between the ratio of mass changes $M_{t}/M_{max}$ and the diffusion coefficient D can be derived [15].

(8) $$\frac{M_{t}}{M_{max}}=\frac{2}{d}\sqrt{\frac{D\;t}{\pi }}$$

Equation (8), based on the above assumptions of one-dimensional diffusion, only considers mass transport in the thickness direction. This is a reasonable assumption for thin films but cannot be made for thicker specimens. In reality, water absorption takes place over all surfaces $\;A_{total}\;\left[m^{2}\right]$ of the rectangular samples. The thicker the sample under consideration, the more the side surfaces influence the diffusion behaviour. For this reason, a modification of Equation (8) is implemented to consider all side surfaces.

(9) $$j=\frac{M_{max}}{V}\cdot \sqrt{\frac{D}{\pi \;t}}$$

The number of particles per area and time unit is called particle flow $j\;[\%/\left(m^{2}t\right)$]. For the initial area of sorption, it can be determined by deriving $M_{t}$ in Equation (8) according to the time t and the considered surface and by applying Equation (7). In the initial stage of sorption $M_{t}/M_{max}=0.5$, it can be assumed that the diffusion profiles on the different sides of the sample do not influence each other and therefore develop independently to each other. The particle flow j, in each case perpendicular to the associated surface, is thus the same in all directions. If the influence of all of the six side surfaces of the sample on the time-dependent water absorption is to be considered, the particle flow must be integrated over the entire sample surface $\left(A_{total}=\overset{6}{\underset{1}{\sum }}A_{i}\right)$. Time integration and a transformation according to the ratio of the mass change results in:

(10) $$\frac{M_{t}}{M_{max}}=\frac{2\;A_{total}}{V}\sqrt{\frac{D\;t}{\pi }}$$

In Equation (10), all side surfaces and therefore the ratio of surface area to volume of a sample to be observed are considered during the diffusion process.

Particularly in the case of thick test specimens, it is obvious that the sorption process in atmospheric conditions takes place over long periods of time and as a consequence the properties change continuously. Furthermore, the equations illustrate that Fick’s diffusion behaviour results in a linear dependence of the mass change ratio $M_{t}/M_{max}$ on the square root of time $\left(\sqrt{t}\right)$, which is also referred to as Case I Diffusion [29].

### 2.2. Calculation Basics on the Swelling Process

During the sorption process, an increase in volume or swelling of the material in all three dimensions can be observed. The reason for this is the increased space requirement of the diffusing water molecules between the PA6 polymer chains. The percentage change $L_{t}\;\left[\%\right]$ of a test specimen dimension can be determined by means of the following formula:

(11) $$L_{t}=\frac{l_{t}-l_{0}}{l_{0}}\cdot 100$$

Equation (11) uses the initial dimension $l_{0}\;\left[m\right]$ of the dry sample and the dimension $l_{t}\;\left[m\right]$ at the respective time t (see Equation (6)) [23]. If both the percentage mass change $M_{t}\;\left[\%\right]$ and the percentage dimensional change $L{\;}_{t\;}\left[\%\right]$ are plotted over the root time and normalised to their respective maximum values ($M_{max}$ and $L_{max}$), the sorption and swelling curves found in the literature are obtained. It is known from the literature that sorption is linear over root time, whereas swelling has an S-shaped curve [23]. This indicates that the expansion, especially in the initial area, is slower than the increase in mass [30].

The S-shape of the swelling curves can be attributed, in particular, to the extreme concentration gradient within the sample at the beginning of the sorption process. By this time, the bulk material is still completely dry, whereas the boundary areas, which are in direct contact with the water or humidity, are already completely saturated and as a result swell considerably. However, the dry bulk restricts the free expansion of the edge areas so that internal stresses are induced in the material, which restrict the overall swelling of the part [22,23,28,31].

The actual expansion resulting from the swelling of the individual areas can be determined by compensating for the swelling-induced stresses. The analytical procedure is based on the equilibrium of the forces in the test specimen:

(12) $$\underset{A}{\int }F_{A}\;dA+\underset{V}{\int }F_{V}\;dV=0$$

Equation (12) includes both the surface forces $F_{A}\;\left[N/m^{2}\right]$ on the surface of the considered body and the volume forces $F_{V}\;\left[N/m^{3}\right]$ within the sample. The forces result from internal stresses $\sigma \;\left[N/m^{2}\right]$ (compressive and tensile stresses) in the specimen, which can be calculated using Hooke’s law for small linear elastic total deformation. The swelling process results in a change of volumetric strain parts, which are characterised by the bulk modulus. As shown in [32], the change of volumetric strain can be assumed to be approximately constant for many thermoplastic polymers and small prevailing elongations. Furthermore, the moisture-induced swelling process and the resultant elongation are assumed to be independent of time or strain rate.

(13) $$\sigma =E\cdot \left(\varepsilon_{ges}-\varepsilon_{f}\right)$$

In Equation (13), $E\;\left[N/m^{2}\right]$ is the Young’s modulus of the material, $\varepsilon_{f}\;\left[\%\right]$ is the strain caused by the swelling of the material, and $\varepsilon_{ges\;}\left[\%\right]$ describes the resulting total strain. If the resulting elongation $\varepsilon_{ges\;}\left[\%\right]$ of each individual area is known, the swelling due to water absorption $\varepsilon_{f}\;$can be determined [23].

### 2.3. Overview of Modelling the Sorption and Swelling Behaviour

The water absorption of PA6 and its influence have already been discussed in a large number of publications. Abacha et al. [15] and Vlasveld et al. [14] each studied the influence of water absorption on the mechanical properties of pure and fiber-reinforced PA6 at different temperatures. The studies differed, for example, in the choice of the sample geometry since Abacha et al. [15] focused on bar-shaped specimens whereas Vlasveld et al. [14] examined injection-moulded sheets. But both aimed to determine diffusion coefficients from the experimental data. These were calculated on the basis of Fick’s law from the initial slopes of the sorption curves. Abacha et al. [15] determined the diffusion coefficients as a function of the thickness of the test specimen (see Equation (8)), while Vlasveld et al. [14] used the ratio of volume to surface area (V/A ratio) (see Equation (10)). In addition, in both publications, temperature dependence was described using a modified Arrhenius equation.

In addition to the different approaches of determining the constant diffusion coefficients discussed in the literature, the calculation of the complete sorption process, including the range of saturation of the sorption process, was also considered in various publications. Arhant et al. [33] calculated the water absorption of PA6 samples in seawater initially on the basis of Fick’s diffusion. Using constant diffusion coefficients in direct comparisons with experimental data sets, it was found that constant diffusion coefficients can only be assumed at the beginning of the sorption process. At higher water concentrations, nonFickian behaviour developed, and the diffusion coefficient increased. This was explained by the water-induced reduction of the glass transition temperature $T_{g}$ and was considered for water temperatures of 15 °C to 40 °C. An FE model was developed that considered this decrease of $T_{g}$ locally and determined the diffusion coefficient depending on this decrease. If the water bath temperature was below $T_{g}$, the diffusion coefficient was determined using a modified Arrhenius equation. If $T_{g}$ was above the water bath temperature, the diffusion coefficient was calculated according to an approach by Vrentas et al. [34,35] and Hong [36] using the free volume theory. Using the method developed by Arhant et al. [33], good agreement between experimental data and numerical calculation was obtained.

Further publications also discussed the determination of concentration-dependent diffusion coefficients and a deviation from Fick’s diffusion behaviour at increased water concentrations, so it can be assumed that this is not negligible [16,23,37]. Joannès et al. [38] provided an approach for the consideration of concentration-dependent diffusion coefficients. Although they focused on the water absorption of fiber-reinforced polyphthalamide (PPA), they provided an approach that can be transferred partially to pure PA6. The applied finite difference model used both temperature- and concentration-dependent diffusion coefficients, which were determined from experimental data via non-linear regression.

Monson et al. [16] studied the analytical calculation of the moisture-induced swelling of various polyamide sheets in water. The calculation model was based on a Fourier series solution of Fick’s 2nd law and used constant diffusion coefficients. These were determined from the initial slope of experimentally determined sorption curves (see Abacha et al. [15]). The swelling calculation was carried out separately for all three spatial directions and differed from the sorption calculation solely by the ratio of the third root. Using this approach, it was not possible to calculate the range of saturation in the sorption process and the characteristic S-curve of swelling. Therefore, the method presented by Monson et al. [16] was only considered as an estimation. It was pointed out that a numerical calculation with concentration-dependent diffusion coefficients and a compensation of the swelling-induced stresses appeared to be appropriate.

The FE model developed by Sharma et al. [37] combined the diffusion and swelling of PA6 sheets in water combined with the properties of a viscoelastic material. The sorption calculation was based on Fick’s laws with concentration-dependent diffusion coefficients. The latter were determined via experimental data using the optimisation algorithm of Nelder and Mead [39]. The swelling analysis, on the other hand, was based on a linear relationship between moisture-induced expansion and concentration and was not able to model the S-shaped course of swelling.

The approach of Inoue and Hoshino [23], which was also based on experimental data, was however able to reproduce both the last stages of sorption and the S-course of swelling of thin PA6 films. For this purpose, several sorption and desorption curves of different ambient humidity levels were measured, and their diffusion coefficient was determined on the basis of the respective slope. Subsequently, a concentration dependence was derived from the saturation values of the curves. In addition, the moisture-dependent Young’s modulus and the maximum linear expansion in the respective saturation state were considered in order to establish the dependency of concentration on these variables as well. In a first step, the finite difference method used calculated the concentration distribution in the sample based on Fick’s laws. On this basis, the moisture-induced strains were determined and the total degree of swelling of the sample was deduced by means of a stress compensation, as proposed by Monson et al. [16]. The publication was limited exclusively to a one-dimensional observation of the specimen in the length direction.

The aim of this work was the experimental analysis as well as the numerical calculation of the sorption and swelling behaviour of geometrically simple test specimens of PA6 in water. The investigations were be carried out at elevated temperatures to imitate common conditioning methods using a water bath. Furthermore, the influence of different specimen geometries and component areas on the sorption and swelling process were analysed. For this purpose, a step-shaped component geometry with different wall thicknesses or volume to surface ratios (V/A ratio) was developed. This step-shaped component was exposed to different humidity environments until weight and volume constancy was achieved to obtain an experimental data basis for the sorption and swelling curves as a function of the surrounding water concentration. The range up to reaching the maximum water absorption as well as the maximum volume expansion were of particular interest here, and were determined using comparably simple and practicable measuring methods. On the basis of the experimentally determined data, the numerical calculation of the sorption and swelling behaviour combining the approaches of Inoue and Hoshino [23] and Vlasveld et al. [14] was carried out with the help of the simulation environment software Abaqus by Dassault Systèmes.

## 3. FE Modelling of the Sorption and Swelling Behaviour

### 3.1. Introduction of the FE Model Used

**Figure 2:**
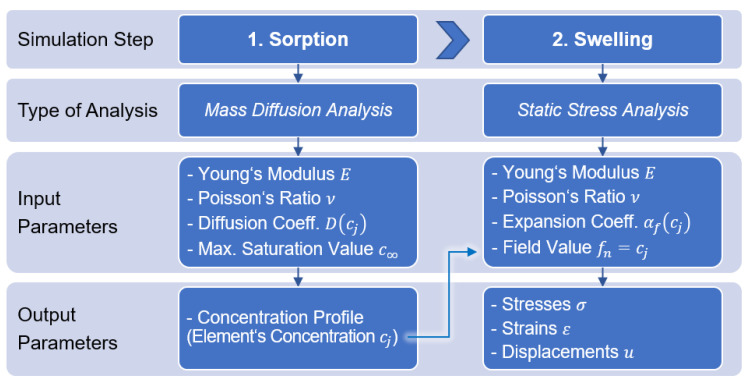
Schematic representation of the successive simulations to calculate the sorption and swelling behaviour of PA6 in water using Abaqus.

The simulation environment software Abaqus from Dassault Systèmes (France) can be used to calculate the sorption and swelling behaviour of PA6 in water. This software, however, does not offer the possibility of coupled calculation of sorption and swelling in the same analysis. The processes of moisture absorption and swelling are known to be a physically coupled system. For the numerical simulation of this coupled process, a simplification must be made for Abaqus that uses a separation of the water diffusion and the swelling process within the simulation environment. Therefore, two successive analyses have to be performed, as shown in Figure 2. First, the sorption behaviour must be determined by using a mass diffusion analysis, followed by a static stress analysis to calculate the water-induced swelling behaviour.

The mass diffusion analysis allows the calculation of a time-dependent concentration profile of the specimens of interest using concentration-dependent diffusion coefficients at constant ambient conditions. The time-dependent concentration profiles can be transferred to the integration nodes of the finite element mesh of the following static stress analysis by means of field variables so that the local concentration is considered at every discretized point in time and space. At the integration points of the FE mesh, each concentration is associated with a corresponding Young’s modulus, a Poisson’s ratio, and three direction-dependent strain values in order to describe the principles of the static stress analysis. The static stress analysis is used to calculate the concentration-related stress and deformation profiles of the specimen. Assuming a reversible water-induced expansion of PA6, an elastic material model is used. However, this does not include irreversible effects, such as hydrolysis or post-crystallisation or re-crystallisation, which could be caused by the water in the material and must therefore be regarded as a simplification. In addition, there is a one-way coupling of the two analysis methods: the results of the mass diffusion analysis influence the static stress analysis, but this has no reverse influence on the diffusion analysis [40,41].

This study was based on the approach of Inoue and Hoshino in [23] as their method considered both the implementation of concentration-dependent diffusion coefficients and the equalisation of swelling-induced stresses. These two aspects seem to be indispensable for the calculation of the characteristic sorption and swelling behaviour of polyamides since there is good agreement between experimentally determined and calculated data using the Inoue and Hoshino approach. In addition, the above-mentioned approach assumes that stress relaxation has almost no influence on the swelling behaviour of the material and therefore does not have to be considered in the calculation, nor is a restriction of the temperature range provided. However, a one-dimensional consideration of the diffusion processes is made, hence the mentioned approach is limited to thin films. The intention of the present study was to simulate the sorption and swelling behaviour of parts with wall thicknesses in the millimetres range, so the consideration was extended to three dimensions and calculated using the simulation environment software Abaqus.

For this purpose, different approaches for determining the diffusion coefficients known in the literature were first considered and then examined for their usefulness. In this context, the consideration of the volume/surface ratio (V/A ratio) of the test specimens, as proposed by Vlasveld et al. [14], was applied.

By means of a geometry with several areas of varied V/A ratios developed particularly for the research question, test specimens were injection moulded and used for the experimental determination of the sorption and swelling curves. Based on the experimental data, numerical calculations were performed and their results were analysed.

### 3.2. Modelling of the Sorption Behaviour Using Mass Diffusion Analysis

To calculate the sorption behaviour, the mass diffusion analysis provided in Abaqus was used based on the fundamentals of Fick’s laws. The analysis model allows the option of Fick’s diffusion or diffusion based on pressure or temperature gradients and is also suitable for representing the nonuniform solubility of the diffusing substance in the base material. The mass diffusion analysis is based on the mass conservation and is calculated by considering the concentration of the diffusing substance c [ppm] and the concentration flux J [m⁄s] for a volume $V\;\left[m^{3}\right]$ with the surface $A\;\left[m^{2}\right]$ with the normal vector n [−] perpendicular to the surface in Equation (14).

(14) $$\overset{\;}{\underset{V}{\int }}\frac{dc}{dt}dV+\overset{\;}{\underset{A}{\int }}n\cdot J\;dA=0$$

Using the Gauss integral theorem, Equation (14) can be transformed into a volume integral so that Equation (15) is valid.

(15) $$\overset{\;}{\underset{V}{\int }}\left(\frac{dc}{dt}+\frac{\partial }{\partial x}\cdot J\right)dV=0$$

To calculate the concentration, a solubility variable ϕ [ppm] is defined, which is referred to as the activity of the diffusing material and describes the degrees of freedom of the network nodes. It is calculated by means of Equation (16) from the mass concentration c [ppm] of a substance and its solubility s [−] in the material of interest. The solubility variable ϕ, as a sort of normalised concentration, enables a continuous calculation of the solubility across the interfaces of different materials as well [40,41].

(16) $$\phi =\frac{c}{s}$$

Using the definition of the solubility variable, the concentration flux J can be calculated from Fick’s 1st law, resulting in Equation (17) shown for calculating the concentration flux with the aid of the Equation (1).

(17) $$J=-D\cdot \left(s\frac{\partial \phi }{\partial x}+\phi \frac{\partial s}{\partial x}\right)$$

If the simplification is adopted that only homogeneous base material is considered, the solubility s is to be regarded as constant and Equation (17) can be summarised as shown in Equation (18) [40].

(18) $$J=-sD\cdot \frac{\partial \phi }{\partial x}$$

In order to ensure differentiability in the solution of the differential equation from Equation (17) for the finite element method used, the variable δϕ [ppm] is added for the weak formulation of the equation related to the respective boundary value problem using Equation (19).

(19) $$\overset{\;}{\underset{V}{\int }}\left[\delta \phi \left(\frac{dc}{dt}\right)+\frac{\partial }{\partial x}\cdot \left(\delta \phi \;J\right)-J\cdot \frac{\partial \;\delta \phi }{\partial x}\right]dV=0$$

The variable δϕ [ppm] describes a continuous scalar field. By inserting the Equation (18) to calculate the concentration flux J and applying the Gaussian integral theorem again, Equation (19) is converted into Equation (20).

(20) $$\overset{\;}{\underset{V}{\int }}\left[\delta \phi \left(s\frac{d\phi }{dt}\right)+\frac{\partial \;\delta \phi }{\partial x}\cdot sD\cdot \frac{\partial \phi }{\partial x}\right]dV=\overset{\;}{\underset{A}{\int }}\left[\delta \phi \;n\cdot \left(sD\cdot \frac{\partial \phi }{\partial x}\right)\right]dA$$

For the time-dependent representation of the diffusion processes of water in PA6, transient states are considered with the help of mass diffusion analysis. For the time integration of a transient state, Abaqus uses the implicit Euler method (“Backward Euler Method”). With the interpolation functions of the form $N^{N}$, the normalised concentration field δϕ can be determined, resulting in the following relationship in Equation (21).

(21) $$\delta \phi =N^{N}\;\delta \phi^{N}$$

In the equation, $\delta \phi^{N}$ now represents a discrete value. To ensure the continuity of the model, nodes of neighbouring elements are shared for discrete calculation, resulting in the discrete formulation:

(22) $$\overset{\;}{\underset{V}{\int }}\left[N^{N}\left(s\frac{\phi -\phi_{t}}{\Delta t}\right)+\frac{\partial N^{N}}{\partial x}\cdot sD\cdot \frac{\partial \phi }{\partial x}\right]dV=\overset{\;}{\underset{A}{\int }}\left[N^{N}\;n\cdot \left(sD\cdot \frac{\partial \phi }{\partial x}\right)\right]dA$$

A non-linear system is obtained once the diffusion coefficient D is dependent on the concentration c. Due to its asymmetric system of equations, this is now solved by an asymmetric solution scheme in Abaqus [40,41].

Fick’s diffusion behaviour with isotropic diffusion properties was selected in the material model. In addition, the diffusion coefficients were defined as concentration-dependent with complete solubility (s=1) of the diffusing agent in the material so that the solubility variable ϕ corresponded exactly to the concentration c according to Equation (16). For a full definition of the material model used, the Young’s modulus and the Poisson’s ratio were also defined, however, these were irrelevant for the calculation of the mass diffusion analysis. A constant water concentration on the surface of the specimen was applied as a boundary condition. The water concentration applied corresponded to the respective maximum value of the sorption curve in ppm.

### 3.3. Modelling of the Swelling Behaviour Using Static Stress Analysis

To calculate the water-induced swelling behaviour of PA6, a static stress analysis was performed in Abaqus based on the results of the previously described mass diffusion analysis and was performed subsequent to it. In the analysis, field expansion was used, which assigns strain values to the material as a function of temperature T and/or a field variable $f_{n}$. This field expansion $\varepsilon^{f}\;\left[\%\right]$ can be defined by means of an expansion coefficient $\alpha_{f}$ and a reference value $f_{n}^{0}$ whose dimension depends on the field variable and can be implemented both isotropically and orthotropically as well as anisotropically. An isotropic field expansion depends on a single expansion coefficient, whereas an orthotropic field expansion requires three coefficients $\left(\alpha_{f11},\;\alpha_{f22},\;\alpha_{f33}\right)$, one for each of the three main directions. The anisotropic field expansion, on the other hand, is determined with the help of six expansion coefficients. Neglecting a temperature dependence that is not considered further in the following equations, the relationship shown in Equation (23) results in a single field variable and the isotropic field expansion [40].

(23) $$\varepsilon^{f}=\alpha_{f}\left(f_{n}-f_{n}^{0}\right)$$

**Figure 3:**
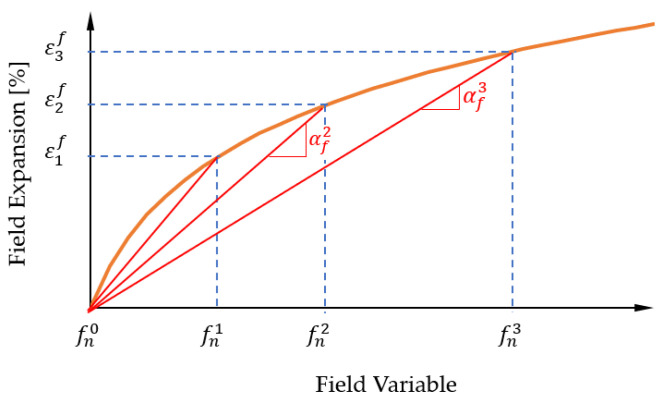
Determination of the expansion coefficient [40].

Here, the strain $\varepsilon^{f}$ to be applied is calculated from the current value of the field variable $f_{n}$, the expansion coefficient $\alpha_{f}$, and the reference value $f_{n}^{0}$ and must be determined for the different direction-dependent expansion coefficients in the orthotropic case. Since the relationship between the field variable and the field expansion is generally known, but not the expansion coefficient to be implemented, this must be determined manually from experimentally determined data. Figure 3 shows the relationship between the field expansion $\varepsilon^{f}$ and the field variable $f_{n}$ used. Here, the respective slope of the connecting line between the reference value $f_{n}^{0}$ at the origin and the function value of the field expansion $\varepsilon^{f}$ ($f_{n}^{0}$), which depends on the field variable plotted on the *x*-axis, provides the respective associated expansion coefficient $\alpha_{f}$.

A field variable was assigned to each element of the FE model. A corresponding field expansion value was calculated for the current value of this variable in each time step with the help of the expansion coefficient, which was then subsequently assigned to the increment. The expansion of the finite element thus imposed generates an (internal) stress $\sigma \;\left[N/m^{2}\right]$ in the material within a structure that cannot be freely expanded.

A static stress analysis carried out in Abaqus is based on the principle of compliance with the equilibrium of forces and moments, so the calculation of the water-induced swelling process in PA6 described in Section 2.2 was valid according to Equations (12) and (13). Since both equations were used in the analysis, no additional subroutines for calculating the material behaviour needed to be implemented in Abaqus. Furthermore, the same settings regarding the geometry of the mass diffusion analysis were used for the static stress analysis so that the concentration profile calculated in the mass diffusion analysis could be transferred to the FE mesh of a static stress analysis.

During the static stress analysis, a normalised concentration value (NNC) is imported from the mass diffusion analysis output file for each element at each time step using the predefined field variables. It is thus possible to include a Young’s modulus as well as a Poisson’s ratio dependent on the field variable NNC in the analysis and thereby influence the incremental strain due to the field expansion. Therefore, water-induced swelling of PA6 can be calculated within the analysis since the field expansion $\varepsilon^{f}$ depends on the current concentration $\left(f_{n}=c_{j}\right)$ of the finite element. The reference value $f_{n}^{0}$ is the initial water concentration $c_{0}\left(=0\;\mathrm{ppm}\right)$ of the dry material.

Since the water-induced swelling of PA6 shows a direction dependence, orthotropic material behaviour was implemented for the FE model based on experimentally determined expansion coefficients $\alpha_{f}$ for the main directions. To investigate the deformation caused by water-induced swelling, material sets are defined in the length, width, and thickness directions of the part under consideration, which can be used to evaluate the calculated simulation results and compare them with experimentally determined swelling data.

### 3.4. Determination of the Relevant Values for the FE Model

The previously presented method for FE modelling of the sorption and swelling behaviour of PA6 in water is based on experimentally determined characteristic values, therefore an overview of the necessary characteristic values and their experimental determination is provided. The diffusion process is significantly influenced by concentration-dependent diffusion coefficients D(c) and limited by the respective maximum saturation concentration $c_{\infty }$ related to the ambient concentration. To determine the input data for the mass diffusion analysis, sorption curves are to be measured at different ambient concentrations at a constant temperature on samples with different volume to surface ratios (V/A ratio). By using different sample geometries with differences in the V/A ratio, a geometry dependence of the sorption can be verified.

For the static stress analysis, material-specific parameters such as Young’s modulus and Poisson’s ratio are required. Since these mechanical parameters are strongly dependent on the moisture content in the material, these parameters are required as a function of the water concentration. In order to ensure that an influence of concentration gradients over the sample cross-section of the results can be excluded, only samples that are completely saturated in the respective ambient concentrations are used for the determination of the mechanical characteristic values as well as the swelling of the material induced by water during the measurement of the expansion. Only in the case of the steady state of complete saturation in the respective ambient humidity the characteristic material values (Young’s modulus and Poisson’s ratio as well as the three direction-dependent expansion coefficients) can be assigned to the respective saturation concentration $c_{\infty }$.

In order to consider a dependence of the experimentally determined data on the concentration c in Abaqus, the sorption curves related to the mass change in percent M(t) must be converted into concentration-dependent quantities. This conversion is performed by using the density of the material $\rho_{PA6}\;\left[\mathrm{kg}/m^{3}\right]$ and the density of water $\rho_{H_{2}O}\;\left[\mathrm{kg}/m^{3}\right]$ at the considered ambient temperature T [°C] and can be calculated with Equation (24):

(24) $$\begin{array}{c} c\left(t\right)=M\left(t\right)\cdot \frac{\rho_{PA6}\left(T,\;t\right)}{\rho_{H_{2}O}\left(T\right)}\approx M\left(t\right)\cdot \;\underbrace{\frac{\rho_{PA6}\left(T\right)}{\rho_{H_{2}O}\left(T\right)}\;}\\ \;\;\;\;\;\;\;\;\;\;\;\;\;\;\;\;\;\;\;\;\;\;\;\;=const.\;\left(for\;T=const.\right) \end{array}$$

In the context of this study, the temperature-dependent density was derived from the pvT behaviour of PA6 taken from the materials data sheet. However, it should also be mentioned at this point that the density is locally influenced by the process-specific morphology of the plastic and its (local) moisture content. The latter is not usually taken into account in the determination of the pvT behaviour and is also not considered within the investigations of this contribution.

The selected boundary conditions of the mass diffusion analysis are derived from the maximum saturation value for the respective sample environment, which is both concentration- and temperature-dependent, and is determined on the basis of the maximum value of the respective sorption curve. For this purpose, it is also necessary to convert the percentage mass increase into the concentration present as well as for the comparison of simulation results and measurement results. Here, a reverse calculation of concentration-related values of the simulation into mass-related values of the experimentally determined data is necessary. However, since a constant conversion factor is assumed due to the simplification described, the conversion has no effect on the quality of the results.

For the FE model described in Section 4.2, sorption curves are thus measured at different ambient humidities as well as in the water bath at constant temperature in each case using various test specimens with different V/A ratios. Using these sorption curves, concentration-dependent diffusion coefficients and the maximum concentration-dependent saturation can be determined. In addition to the concentration-dependent sorption curves, concentration-dependent swelling curves are also required, which are used to determine the concentration-dependent expansion coefficients of the three spatial directions. Finally, concentration-dependent Young’s moduli are measured in the uniaxial tensile test using the fully saturated samples at different ambient humidities, which are converted into an approximation of the the corresponding concentration-dependent Poisson’s ratios using Equation (25), according to Gienke and Meder [42].

(25) $$\upsilon^{*}=0.3+0.2\left(1-\frac{E^{*}}{E_{max}}\right)$$

The equation calculates the Poisson’s ratio $\upsilon^{*}\;\left[-\right]$ of the desired material condition from the corresponding Young’s modulus $E^{*}\left[N/{\mathrm{mm}}^{2}\right]$ and the maximum Young’s modulus $E_{max}\;\left[N/{\mathrm{mm}}^{2}\right]$ of the respective material. According to the data sheet for the polyamide used, $E_{max}$ is $3500\;N/{\mathrm{mm}}^{2}$ [24].

## 4. Materials and Experimental Approaches

### 4.1. Introduction of the Developed Specimen Geometry

**Figure 4:**
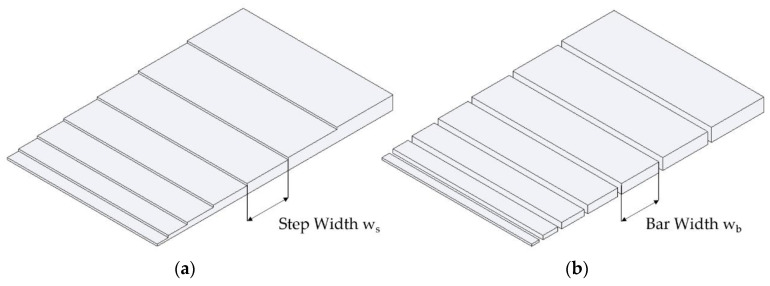
Step shaped part geometry (**a**) and bar-shaped samples cut from step part (**b**).

To ensure that the influence of different part wall thicknesses was considered for determining the sorption and swelling behaviour, a simple, step-shaped sample geometry was developed. This geometry has the advantage that it represents a part with different wall thicknesses, which can also be separated at the steps into individual bar-shaped parts. The sample geometries are shown in Figure 4. The step-shaped part is shown on the left, whereas the single (bar) samples with differing dimensions cut from the step-shaped geometry are shown on the right side.

By separating the steps, individual, cuboidal test specimens (bars) were received, which allowed a specific interpretation of the physical behaviour as well as the influence of geometry. The knowledge gained from the individual bar samples was used to build the FE model, whereas the complete step-shaped part was used for verification. Additionally, conclusions can be drawn about the interaction of the individual part areas.

The dimensions and V/A ratios for the step-shaped part and the individual samples are listed in Table 1.

The step-shaped specimens were produced in an injection moulding process on an Arburg 370 S (Arburg, Germany) using the material Durethan B 31 SK (Lanxess, Cologne, Germany), details of its properties and processing parameters can be found in the data sheet [24]. The part design and the balancing of its gating system was based on the design guidelines of Beaumont [43] and Hopman et al. [44] and was optimised using the injection moulding simulation software Autodesk Moldflow (Autodesk Inc., San Rafael, CA, USA).

After injection moulding, the mouldings were separated from their sprue system. One half of the moulded part was cut into individual bar samples with a band saw and deburred using a scalpel. The step-shaped test specimens and the individual bar samples were subsequently annealed for 100 h at 70 °C in a vacuum furnace (Heraeus Holding GmbH, Hanau, Germany) so that a post-crystallisation could take place before the start of the test and the respective dry weights $m_{0}$ or dry dimensions $l_{0}$ of all samples could be determined using precision balance (HR-250A, A&D Company, Tokyo, Japan) with a resolution of 0.0001 g.

### 4.2. Determination of Implemented FE Model Parameters

The measurement of the sorption and swelling curves was performed on the developed step-shaped part as well as on the individual cut bar specimens with different geometries. Ten identical test specimens (bar samples and step-shaped parts) were examined. The individual samples were analysed together, resulting in a total of 70 specimens per ambient condition. For this purpose, the test specimens were stored in a water bath (Emmi 40 HC, EMAG, Germany) as well as in a climate chamber (SB1/300/40, Weiß, Germany) at different relative humidities (90% r.h./60% r.h.) until complete saturation. The sorption and swelling curves of the samples stored in the water bath were required for the analysis of the water absorption, whereas the sorption and swelling curves determined at different relative humidities in the climate chamber were only needed for the definition of the FE model. After reaching the respective saturation state, the Young’s modulus of the respective individual samples was also determined. The step-shaped parts were saturated in the water bath solely for the determination of sorption and swelling behaviour.

The water was absorbed at a temperature of 80 °C. This temperature was chosen because, on the one hand, it distinctly accelerates water absorption compared to saturation at room temperature and, on the other hand, it is significantly above the glass transition temperature for dry polyamide ($T_{g}\;$≈ 60 °C) [4].

The sorption curves were determined by their weight increase due to water absorption. A precision balance was used for the measurements. Depending on the ambient conditions, either a water bath or a climatic chamber was used to constantly adjust the sorption conditions. The samples were each stored on perforated grids to ensure that the contact area of the samples with the surrounding medium was maximised. Before each weighing process, all samples were cleaned with a lint-free cloth to remove any water that may have adhered to the surface.

**Figure 5:**
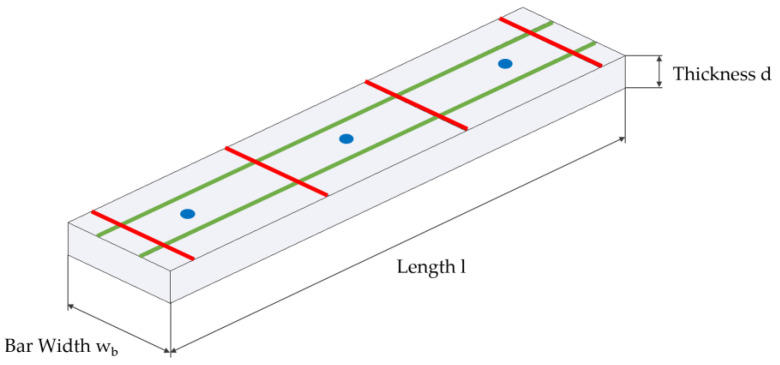
Measurement points for the test specimens, green tactile, red, and blue determined using a digital microscope.

The swelling behaviour was determined in each case after weighing the test specimens by determining the dimensional change from the initial dimensions. The entire swelling behaviour was relevant only in the case of samples stored in a water bath; in the case of samples stored in a climatic cabinet, only the dry dimensions (as a reference value) and the maximum values of swelling after the respective water saturation were determined. The sample thickness was determined on a granite weighing table and was measured tactile by means of a mechanical dial gauge, which was previously calibrated on a gauge block of known thickness. The thickness of the individual samples was determined and averaged at three different points. The measurement of the sample length as well as the sample width was carried out with the help of a digital microscope (DSX500, Olympus, Japan) and was measured at previously determined distances or points (see Figure 5). Length and width were measured at two and four distances (for bars) or four distances (for parts) and averaged.

A similar procedure was also used for the step-shaped samples: the different step thicknesses were measured by using a dial gauge, then the complete length of the steps over the back was determined, and, finally, clamped between two gauge blocks, the respective step widths of the parts were measured (see Figure 4b). The data obtained using this method enabled swelling curves to be recorded in the thickness, width, and length direction depending on the present geometry.

The determination of the concentration-dependent Young’s modulus was performed in the saturated state of the respective environmental conditions. For this purpose, quasi-static tensile tests were carried out at a temperature of 80 °C using an Eplexor 500 N equipped with a humidity generator (Netzsch/Gabo, Selb, Germany). During the measurement, the hygrometer ensured that the same environmental conditions were maintained during the sorption. For the samples stored in a water bath, a relative humidity of 95% r.h. at 80 °C ambient temperature was selected, which represented the maximum possible relative humidity to be set for the device.

The measurement of the Young’s modulus was carried out on three completely saturated individual samples of geometry T1.5 and T2.5 of each moisture-dependent measurement series and additionally on three completely dry bar samples of the same geometry. In order to ensure the appropriate ambient conditions, a soak time of 120 s was applied before starting the respective measurements, during which the set temperature or humidity values may have fluctuated by less than 1%. As the swelling process is globally a very slow process, the E moduli were determined at a traversing speed of 0.1 mm/min and were carried out up to a nominal elongation of the test specimen of 1%. According to DIN EN ISO 527 [45], this elongation is sufficient to determine the Young’s modulus.

## 5. Results and Discussion

### 5.1. Experimental Characterisation of Water Absorption and Swelling

**Figure 6:**
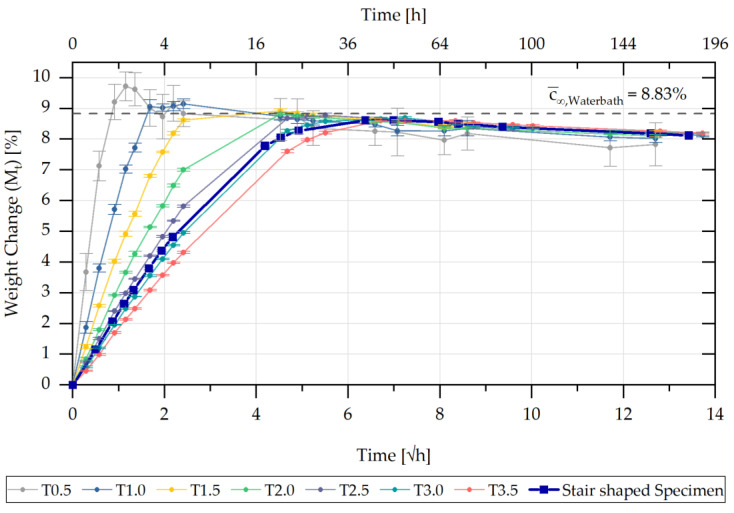
Sorption curves of the different bar specimens and the step-shaped specimen stored in a water bath at 81 °C.

The sorption behaviour of the sample geometries used was measured up to ten days in the various ambient humidities at an average temperature of 84 °C until complete saturation. The percentage change in mass M(t) was observed over the respective exposure time. Ten test specimens for each of the seven different bar geometries were aged in a climatic oven at a humidity of 60% r.h. and 90% r.h. The ten parts of the developed step geometry were additionally completely saturated in a water bath. Using the respective sorption curves, a mean maximum saturation related to the respective ambient concentration was determined. The averaged sorption curves for the seven different bar geometries and the step-shaped parts during storage in the water bath are shown in Figure 6.

**Figure 7:**
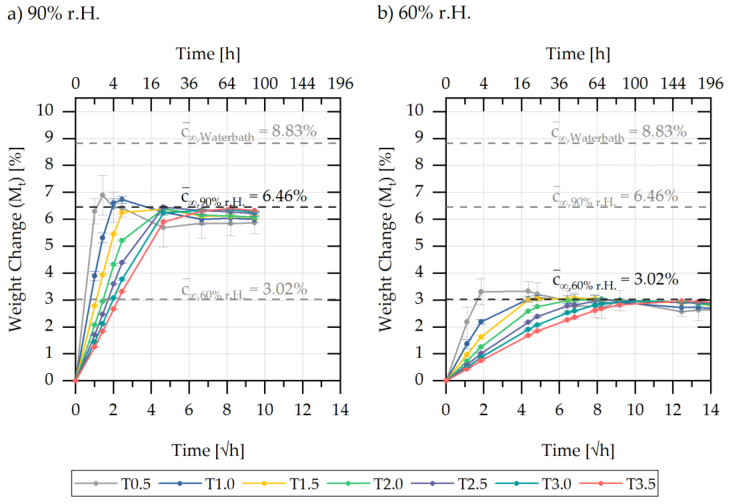
Sorption curves of the different bar specimens and the step-shaped specimen stored in (**a**) 90% r.H and (**b**) 60% r.h. in a climate chamber at 84 °C.

Overall, in the initial time range of the sorption curve (up to M(t) ≈6.5%), a linear dependence of the mass increase M(t) with the root time could be detected, which is related to Fick’s diffusion behaviour and can be explained by the saturation of the polar amide groups by water molecules [1]. In further times, the sorption curves approached a maximum value $M_{max}$, which was reached faster with a lower V/A ratio of the sample geometry. From the maximum values $M_{max}$ of the sorption, the average saturation concentration ${\bar{c}}_{\infty }{}_{Water\;bath}\;\left(=8.83\;\%\right)$ was determined. After reaching maximum saturation, the sample weight decreased slightly. The saturation behaviour, which depends on both geometry and concentration, can also be observed for sorption curves of lower concentrations at 60% and 90% r.h. in Figure 7.

The figure shows the sorption behaviour of the bar specimens in the climatic chamber at relative ambient humidities of 90% r.h. (a) and 60% r.h. (b) at 81 °C ± 1.0 °C. For both series of measurements, the mean saturation concentration ${\bar{c}}_{\infty }{}_{90\%\;r.H.}\left(=6.46\;\%\right)$ and respective ${\bar{c}}_{\infty }{}_{60\%\;r.H.}\;\left(=3.02\;\%\right)$ was determined and increased with the increase of the ambient water concentration during sorption. A weight loss after reaching maximum saturation can be recognised for both sorption curves shown in Figure 7. However, this weight loss was comparatively smaller the lower the relative ambient humidity.

Based on the sorption curves of Figure 6 and Figure 7, it can be derived that there is a geometry dependency in the data: the lower the V/A ratio of the samples, the faster $M_{max}$ is reached and the higher the value of $M_{max}$ as well as the weight reduction after $M_{max}$. This weight reduction is presumably related to additives such as demoulding agents in the PA6 used, as well as to water-induced recrystallisation and will not be considered further for the sorption calculation.

The deviating sorption behaviour of T0.5 could be explained by the influence of the sample production in the injection moulding process. The sample geometry T0.5 was located at the end of the flow path of the injection moulded part and thus farthest away from the gating system, meaning this section of the part was filled last. Due to the small sample thickness of 0.5 mm, there was an increased cooling rate and the lowest holding pressure effect compared to the rest of the part geometry, so a deviating morphology and increased crystallisation degree and/or smaller crystallite size could be assumed [46,47]. These process-induced differences in morphology should be investigated in further work, since an influence on the sorption behaviour seems to be given. Furthermore, the samples of geometry T0.5 showed a comparatively high standard deviation and thus measurement uncertainty, which can be explained by the comparably low sample mass. All sample geometries were measured on the same balance so that the samples T0.5 with the lowest sample weight had the highest signal-to-noise ratio. For these reasons, sample T0.5 will not be considered in the following.

The determination of the concentration-dependent diffusion coefficients can be determined with the help of Fick’s laws. For this purpose, the relationship in Equation (10) is converted using the diffusion coefficient $D\;\left({\bar{c}}_{\infty }{}_{j}\right)$ and the V/A ratio of the individual geometry to be considered is included. The relationship shown in Equation (26) is used to determine the factor k and is valid in the range of linear sorption increase.

(26) $$D\left({\bar{c}}_{\infty }{}_{j}\right)=\frac{\pi }{16}\cdot {\left(\frac{\frac{M_{t}\left({\bar{c}}_{\infty }{}_{j}\right)}{M_{max}\left({\bar{c}}_{\infty }{}_{j}\right)}}{\frac{\sqrt{t}}{2\cdot \frac{V}{A_{tot}}}}\right)}^{2}=\frac{\pi }{16}\cdot k{\left({\bar{c}}_{\infty }{}_{j}\right)}^{2}$$

**Figure 8:**
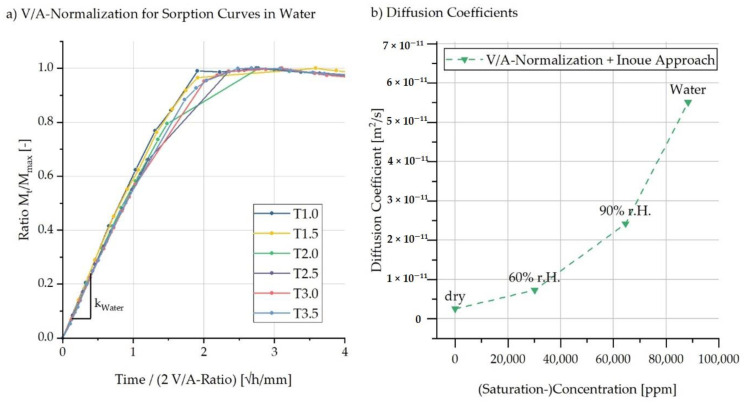
Determination of factor k using V/A normalisation for (**a**) sorption curves in water and (**b**) diffusion coefficients determined by V/A normalisation and the Innoue approach for different (saturation) concentrations.

For the calculation of the concentration-dependent diffusion coefficients from the experimentally determined sorption data, the values of the ordinate $\left(M_{t}\right)$ are normalised to the respective maximum saturation $\left(M_{max}\right)$. Furthermore, the abscissa is normalised to the respective V/A ratio so that the influence of the present sample geometry is subsequently eliminated. The respective diffusion constant corresponds to the slope k of the normalised sorption curves. Figure 8a shows the correlation of the normalised sorption curves with the determination of the diffusion constant for the samples in the water bath at 81 °C.

Since the sorption curves were recorded at different temperature ranges of up to ±5 °C owing to inaccuracies in the temperature control, an Arrhenius approach was used so that all diffusion coefficients can be calculated for the same temperature. This Arrhenius approach exploits the temperature dependence of the diffusion coefficients so that a conversion to 80 °C ambient temperature can be made. The Arrhenius approach is valid for temperatures above the glass temperature ($T_{g}$ ≈ 60 °C) and is given in Equation (27):

(27) $$D\left(T\right)=k\;\cdot e^{\left(-\frac{e_{s}}{RT}\right)}$$

Equation (27) describes the conversion of the diffusion coefficient $D\;\left[m^{2}/s\right]$ for the respective temperature T [K] and uses the general gas constant R=8.3145 J/(mol·K). According to Hanspach and Pinno, the activation energy $e_{s}\;\left[J/mol\right]$ for the diffusion of water in PA6 is 0.49 eV (≙47278 J/mol) [48]. Using the experimentally determined value for the diffusion constant $k\;\left[m^{2}/s\right]$, the temperature-dependent diffusion coefficient D can now be calculated. Figure 8b shows the diffusion coefficients calculated in this way for the different ambient concentrations at 80 °C.

The sorption curves normalised to the V/A ratio shown in Figure 8a indicate that the diffusion coefficients were independent of geometry for the water absorption case in the water bath. Geometry independence was also found for the sorption behaviour at an ambient humidity of 90% and 60% r.h., respectively. This observation was also made by Inoue and Hoshino [23] in their work carried out on thin PA6 films with a normalisation to film thickness for the sorption curves. The authors explained this behaviour by the fact that swelling due to water absorption occurs without a time delay.

A promising approach by Inoue and Hoshino [23] was pursued in the following. In accordance with this, the diffusion coefficient determined by Equation (28) is approximately equal to the integral of the mutual diffusion coefficient $\hat{D}$ over the respective saturation concentration ${\bar{c}}_{\infty }$ multiplied by its reciprocal and the following Equation (28) applies:

(28) $$D\approx \;\frac{1}{{\bar{c}}_{\infty }}\;\int_{0}^{{\bar{c}}_{\infty }}\hat{D}d{\bar{c}}_{\infty }$$

The mutual diffusion coefficient $\hat{D}$ describes the directional diffusion process following the concentration gradient and is determined based on the concentration-dependent change on the product of the determined diffusion coefficient and its respective saturation concentration. Using the mutual diffusion coefficient, the authors obtained a higher agreement between model and measured data, so this approach was applied here. In addition, this method can be used to calculate the self-diffusion of the material in a completely dry state $\left({\bar{c}}_{\infty }=0\;\mathrm{ppm}\right)$. For this purpose, the respective diffusion coefficient is multiplied by its corresponding saturation concentration. A representation of the diffusion coefficients used can be found in Figure 8b.

In the sorption measurements performed, the respective swelling behaviour of all specimen geometries (bars and step-shaped parts) was also measured with the time- and concentration-dependent mass increase. For this purpose, the respective percentage changes in length, width, and thickness related to the dry initial dimensions of the different specimen geometries were determined using Equation (11). The measured swelling curves for the storage of the samples in the water bath are shown in Figure 15 in direct comparison with the swelling simulation performed and are discussed there together (Section 4.2).

**Figure 9:**
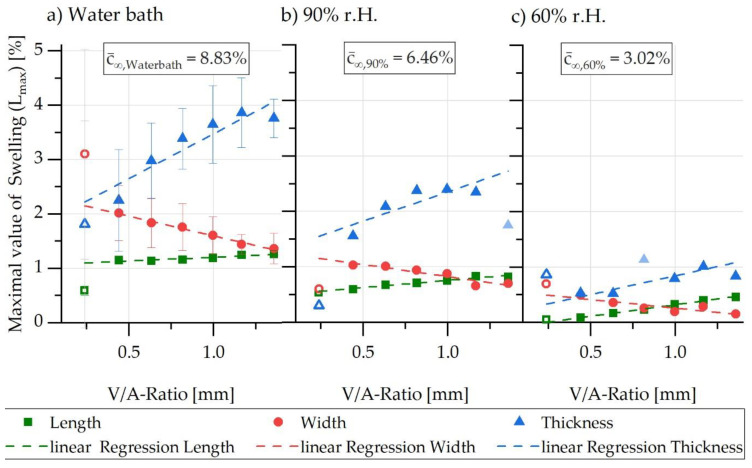
Maximal values of swelling in length, width, and thickness direction for different V/A ratios (**a**) in water bath, (**b**) in 90% r.h. and (**c**) in 60% r.h.

The respective maximum values of the swelling as a function of the sample geometry and swelling direction are shown in Figure 9. Here, a possibility is created by means of linear regression through the respective measured values so that different V/A ratios can be simulated using interpolation between the measured data. According to the ambient water concentration, the respective maximum values of swelling were higher the higher the ambient humidity. In the same way, the changes in percentage of the maximum swelling were most pronounced for the thickness direction, but least pronounced for the length direction. For the length as well as the thickness change, the maximum dimensional change increased for increasing V/A ratios, whereas it decreased for the width direction.

Based on the correlations shown in Figure 9 and the resulting linear regressions, the respective concentration-dependent maximum swells can be extracted for the calculation model and corresponded to the concentration-dependent field expansion $\varepsilon^{f}$ described in Section 3.3 using Equation (23). However, as already described, these correlations are dependent on the V/A ratio of the considered sample geometry as well as on the direction so that orthotropy is assumed for the FE model.

**Figure 10:**
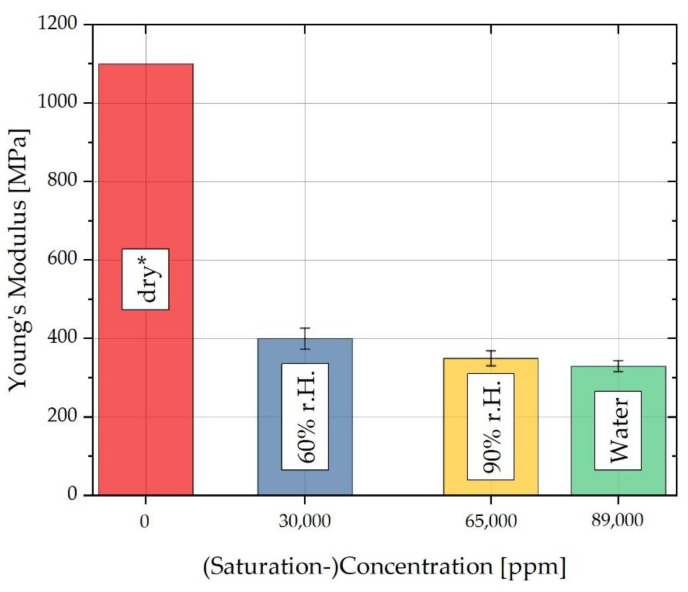
Concentration-dependent Young’s moduli for the different ambient humidities. * The value for dry material is based on data sheet values.

For the internal stress compensation of the water-induced swelling used in the FE analysis, concentration-dependent mechanical parameters in the form of Young’s modulus and Poisson’s ratio are required. For this purpose, uniaxial tensile tests were carried out on the specimen geometries T1.5 and T2.5 with three specimens per geometry and the respective saturation state of the concentrations considered at a strain rate of 0.1 mm/min, as already described in Section 3. The tensile tests were carried out on a Gabo Eplexor500 N with a hygrometer (Netzsch, Germany). The tests were carried out at an ambient temperature of 81 ± 1 °C and 50% r.h. so that the mean temperature of the water bath or the climatic chamber can be replicated. Since the tests were carried out within a few minutes, any effect of redrying on the mechanical characteristics of the test specimens can be neglected. Using the data measured in this way, Young’s moduli were determined, which were converted into corresponding Poisson’s ratios using Equation (25). The values determined for Young’s modulus and lateral contraction are shown in Figure 10.

The values for the concentration-dependent Young’s modulus were supplemented by an iteratively determined Young’s modulus for completely dry material from pre-tests with the help of swelling simulations. This iterative determination was performed because it was the dry Young’s modulus that showed the highest influence on the S-shaped course of the swelling curves. If the elastic modulus for completely dry material was set to 1100 MPa and the corresponding Poisson’s ratio was calculated using this elastic modulus according to the Equation (25), the FE model reproduced the measured swelling curves in a very good approximation. The E modulus values selected for the iterative procedure were taken from the data sheet values of the material used, Durethan B 31 SK [24].

Table 2 presents a summary of the experimentally determined concentration-dependent model variables for 80 °C, which were implemented in the FE simulation environment to determine the water absorption and the swell-induced initial stresses. The expansion coefficients shown in Table 2 are valid for an O/V ratio of 0.44 (corresponding to sample T1.0) and exemplarily selected. The expansion coefficients must be determined using the various direction-dependent linear regression lines from Figure 9 for any O/V ratio.

### 5.2. FE-Based Simulation of Water Absorption and Swelling

Applying the experimental concentration-dependent diffusion constants, a mass diffusion analysis was carried out to model the water absorption using Abaqus. A water concentration $c_{j}=90000\;\mathrm{ppm}\;$was specified as a boundary condition, which corresponds to a part completely surrounded by water. For this analysis, the calculation of the time-related percentage mass change $M_{t}$ for a computation time step is obtained from the quotient of the sum of the dissolved water quantity within the finite elements ESOL and the sum of the respective volumes of the finite elements EVOL of the FE mesh. The amount of dissolved water within an element ESOL is determined from the product of the water concentration in the finite element $c_{j}$ and the finite element volume $V_{j}$:

(29) $$M_{t}=\frac{\sum ESOL}{\sum EVOL}=\frac{\sum c_{j}\left(t\right)\cdot V_{j}\left(t\right)}{\sum V_{j}\left(t\right)}$$

**Figure 11:**
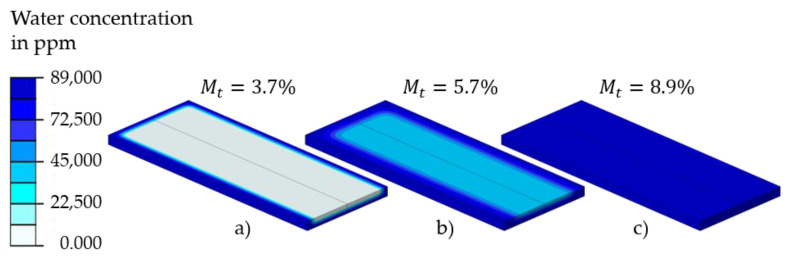
Water concentration of a T2.0 sample for the computation steps: (**a**) *t* = 1.4 $\sqrt{h}$, (**b**) *t* = 2.4 $\sqrt{h}$, (**c**) *t* = 12 $\sqrt{h}$.

The calculation of the local water concentration $c_{j}$ serves as the basis for calculating the total water absorption of a part. This is shown in Figure 11 as an example for the T2.0 specimen. A concentration of $c_{j}=90000\;\mathrm{ppm}$ corresponds to complete saturation with water, whereas the areas with a concentration of 0 ppm are considered as a dry surrounding environment. With increasing water exposure or calculation time, the water diffuses into the specimen and the water concentration in the core of the specimen increases until the saturation state is reached.

**Figure 12:**
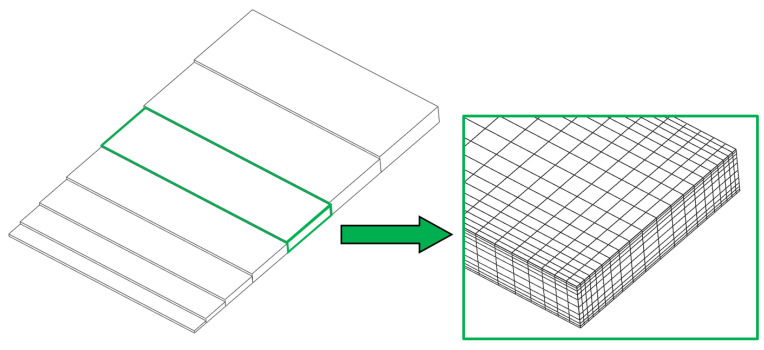
Schematic illustration of the mesh refinement to the boundary layer of a calculated part.

The concentration profiles for selected concentration values in the length, width, and thickness directions clearly showed pronounced gradients from the edges into the core of the sample. Figure 11 also illustrates the boundary layer effects that occur in various circumstances in plastics technology, which means that a finer local discretisation within the FE analysis must also be performed when calculating the diffusion of water into a plastic specimen. This was also illustrated for the FE mesh, using the example of the T2.0 specimen, by increasing the finite element density in the boundary areas of the specimen or part (Figure 12).

In a transient mass diffusion analysis with second order elements, the choice of the first time step is important since a time step chosen too small can cause disruptive oscillations in the solution, which is a numerical problem. Thus, the minimum acceptable time step $\Delta t_{min}\;\left[s\right]$ is determined with the help of Equation (30) [40]:

(30) $$\Delta t_{min}>\frac{1}{6\;D_{min}}\;\Delta l^{2}$$

In Equation (30), $D_{min}\;\left[m^{2}/s\right]$ represents the smallest diffusion coefficient used in the analysis and Δl [m] denotes the finite element edge length of the applied FE mesh. A definition of the time step is required to enable a transfer of the concentration profile of the mass diffusion analysis to the static stress analysis and to ensure the comparability of the results at any time. The time steps are not chosen as constant because the largest concentration gradients occur at the beginning of sorption and therefore the best possible resolution is required. With a sufficiently large diffusion period, the speed of the concentration change decreases so that sufficient resolution can be achieved using larger time steps. Increasing the time steps during the sorption process significantly reduces the calculation time so that a good compromise between calculation time and resolution is achieved.

The transient mass diffusion analysis was performed by using an FE model based on the geometry of the specimen to be analysed. To reduce the calculation time, the symmetry of the sample was considered and a hexahedron mesh of the type DC3D20 was used. This consisted of square elements in order to calculate heat transfer having the optimal edge length Δl determined within a mesh study. The mesh selected by this method was also verified for suitability to be used for the subsequent static stress analysis since, as already mentioned, the applied mesh has to be identical for both analysis steps.

The output variables required from Abaqus to evaluate the results of a transient mass diffusion analysis are the concentration (CONC), the solution variable or normalised concentration (NNC), the element volume (EVOL), and the amount of solute (ESOL). These variables were evaluated using a Python script and the results are subsequently presented as sorption curves.

**Figure 13:**
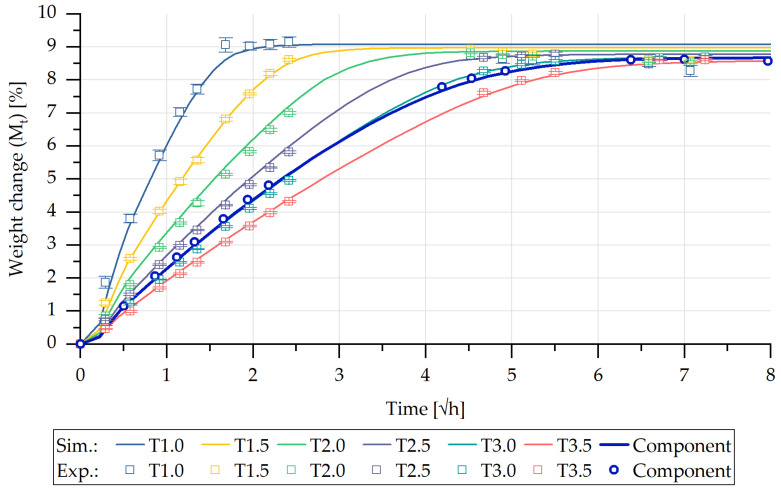
Comparison of the experimentally and computationally determined sorption curves of the individual bar geometries and step-shaped part.

The individual sorption curves could thus be calculated for the seven bar-shaped test specimens, which were taken from the step-shaped injection moulded part, taking into account the corresponding discretisation and evaluation of the simulation results according to Equation (29). Figure 13 illustrates the comparison of the simulated and experimentally determined sorption curves of the rectangular bar specimens.

The computed curves exhibit a correspondence with those of the experimentally determined sorption curves. The larger the volume of the exposed specimen, the longer the period of time needed until the saturation state was reached. The maximum values of the mass changed due to water absorption correspond to those of the experimentally determined values. In contrast to that, Figure 13 also depicts the calculated as well as the experimentally obtained sorption curve of the entire step-shaped part until the saturation state is reached. In this case also, a close correspondence of the sorption curves can be observed. It can be stated that the sorption curves of parts with different wall thicknesses can also be determined using the method described in this contribution.

**Figure 14:**
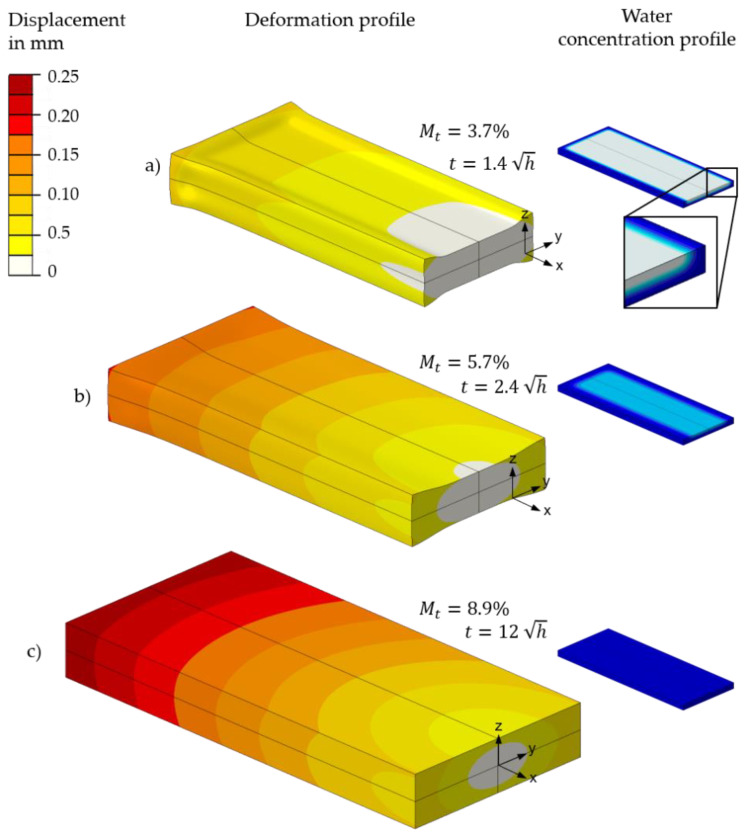
50× enlarged illustration of the swelling-induced deformation for the corresponding water concentration distributions at the computation steps: (**a**) *t* = 1.4 $\sqrt{h}$, (b) *t* = 2.4 $\sqrt{h}$, (c) *t* = 12 $\sqrt{h}.$

In order to analyse the swelling process of a specimen or part, a structural-mechanical analysis was added to the mass diffusion analysis. The locally differing concentration distribution of each computation step was transferred to the structure simulation via a field variable and mapped onto the FE mesh as a predefined field. By implementing concentration-dependent expansion coefficients and stiffnesses in the form of the E modulus, the moisture-induced expansion of each finite element can be determined. In Figure 14, the contour plot of the swelling-induced dimensional change for three selected water concentration distributions is shown on the example of the T2.0 specimen.

The evaluation of the total dimensional change at the part surface in a given direction was carried out in the context of this study by evaluating the sum of the displacements of the individual nodes of the mesh:

(31) $$\bar{U}\left(t\right)=\sum {\bar{U}}_{i}\left(t\right)$$

In an orthogonal consideration of this circumstance, the bar-shaped specimens had different dimensions in the longitudinal, width, and thickness directions so that for the comparison with experimentally determined dimensional changes a normalised representation based on the maximum expansion (in the saturation state) could be used to present the results.

**Figure 15:**
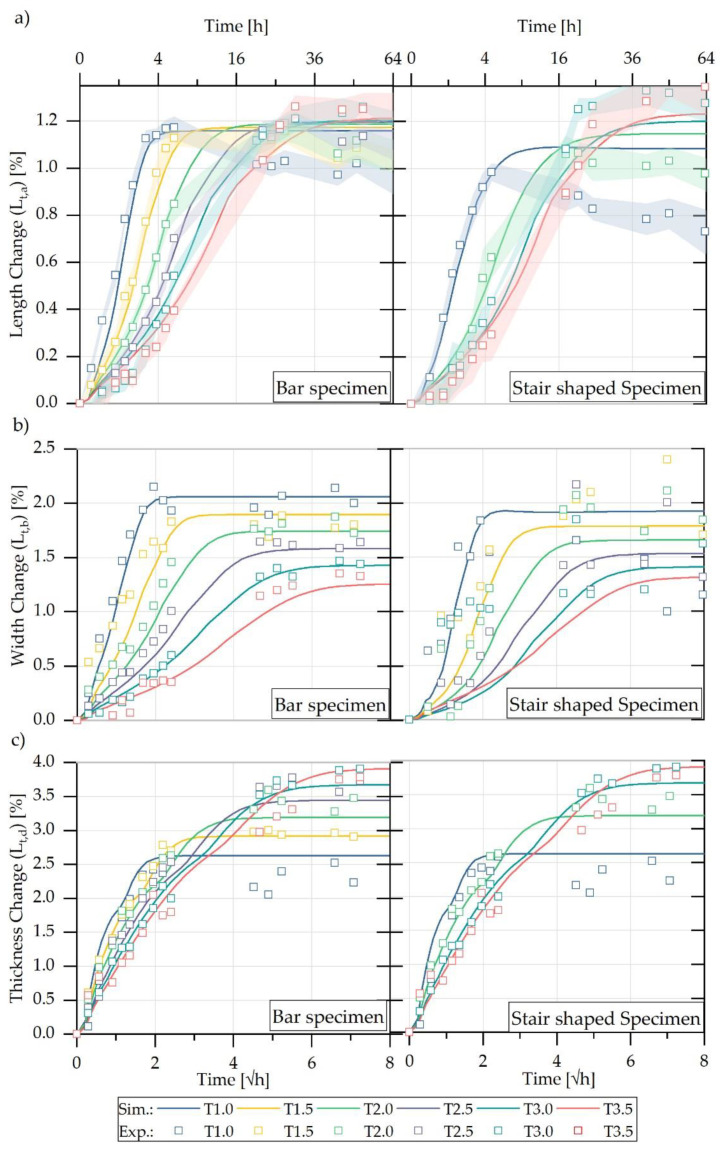
Comparison of the experimentally and computationally determined swelling curves of the different bar geometries and step-shaped part: (**a**) longitudinal, (**b**) width, and (**c**) thickness direction.

For the purpose of comparing the experimental swelling curves presented in Section 4.1, Figure 15 illustrates the percentage dimensional change of the bar specimens related to the initial dimension. The results indicate a good agreement of the curves. Furthermore, the simulation model allowed for the reproduction of the different maximum values of the swelling in the direction of width and thickness.

In the context of the definition of a simplified simulation method for the determination of water absorption and swelling-induced deformation of plastic parts, it was necessary to check whether the swelling behaviour of more complex parts, such as the entire step geometry, can be approximated by simple geometries such as those of the bar test specimens. Figure 15 shows this in a comparison of the individual bar geometries and the corresponding step of the test part. The calculated curves of the steps tended to be lower than those of the bar geometries. This was caused by the mutual influence of the connected steps and the resulting inhibition of the swell-induced expansion. This can be seen particularly clearly in the dimensional change in the width and thickness direction. It should also be emphasised at this point that the characteristic S-shaped course of the swelling curve discussed in the state of the art can be reproduced by the simulation method presented in this contribution. The good correlation of the experimentally and numerically determined swelling curves shows that a reliable calculation of the three-dimensional moisture-induced swelling process of a component can be carried out.

**Figure 16:**
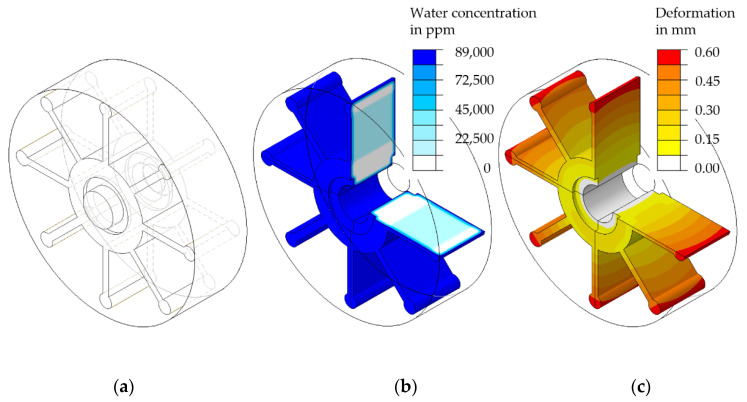
Illustration of a (**a**) pump impeller and its (**b**) calculated water absorption as well as the (**c**) swelling-induced deformation.

Based on this, an orthotropic or transversely isotropic swelling behaviour can be included in the part dimensioning by considering the molecular orientation (e.g., by means of the consideration of flow lines within an injection moulding simulation) and the application of these to the mesh of an FE simulation. However, a wide range of parts have a structure that can be subdivided into simple geometries to which a preferred direction regarding water absorption and swelling can be assigned even without considering a numerically calculated locally different molecular orientation. In the simplest case, a purely isotropic moisture-induced swelling behaviour can be assumed by the user and considered in the computer-aided design of a plastic part. The calculation of the moisture absorption and swelling of a pump impeller is shown here as an example (Figure 16).

The calculation includes orthotropic swelling behaviour and an exposure to water on the surface of the pump impeller. Using the simulation model presented here, the initial stresses of the plastic part can be included in the part dimensioning process in addition to the application-specific loads applied to it. This refers to residual stresses that are caused by locally varying swelling behaviour and are included in the total stress. A further important factor is the possibility of calculating the change in geometric tolerances, which has a direct effect on the performance of a machine part. For the example given here, an influence on the pumping performance would be conceivable. Furthermore, the calculation of a sorption and desorption history is possible so that different moisture contents and the resulting properties of the part and its function within an assembly can be predicted. In future research, the calculation of water absorption and swelling-induced deformation should be validated by means of complex plastic parts and examined for its calculation accuracy. Furthermore, the investigations on moisture absorption need to be extended to the influences of the injection moulding process and the resulting morphological changes.

In order to improve the quality of the evaluation, more precise measurement techniques, such as laser measurement technology [22], moiré interferometry [49], or embedded optical fibres [50] (instead of the tactile measurement techniques used here) have to be used to determine the swelling behaviour of complex parts. In particular, a computer-aided target-actual comparison of the change in form and position tolerances of a part would be ideal. From the perspective of the material-specific properties, aspects related to the influence of the morphological structure (skin-core ratio of injection-moulded parts, different process-related degrees of crystallisation) on the diffusion behaviour need to be investigated experimentally and implemented in the calculation by modifying the diffusion and swelling model.

## 6. Conclusions

The work presented here provides a reliable and practical method for the characterisation and model-based representation of the sorption and swelling behaviour of PA6 in water. The most important results are listed in the following:

- Development of a method for the characterisation and FE modelling of the sorption and swelling behaviour of PA6 parts in water using Abaqus.
- Development of a step-shaped test specimen that can be subdivided into simple bar specimens by means of separation and then characterised individually.
- Using simple characterisation methods, such as the determination of the sorption behaviour based on the mass increase, as well as the swelling behaviour based on the measurement of the dimensional change with microscopy or thickness measurement using a mechanical dial gauge.
- Use of concentration-dependent diffusion coefficients as well as expansion coefficients for FE model development.
- Development of an FE model with good accuracy, which initially separated the coupled processes of sorption and swelling for simplification and calculated them successively.
- Conclusions from the sorption and swelling behaviour of individual sample geometries with different V/A ratios on the part geometry behaviour with varying wall thicknesses.
- Possibility to extend the simulation method to real parts with a complex geometry and wall thickness distribution and also the possibility to extend the method to different surrounding humidities instead of direct contact with water by changing the simulations’ boundary conditions.

Finally, it can be concluded that a reliable simulation method for the consideration of swelling-induced deformation caused by water absorption has been developed within the scope of this study. Swelling-induced deformation can lead to significant residual stresses if it is inhibited or is nonuniformly distributed due to different wall thicknesses of a part. The latter have to be further evaluated for, for example, production-specific initial stresses due to shrinkage processes within the part design. Using the concentration-dependent mechanical properties determined here, this initial stress in a part can be mapped and considered.

## Figures and Tables

**Table 1:** Dimensions of the step-shaped part and the cut bar specimen.

Sample Name	Thickness t (mm)	Length l (mm)	$\mathrm{Step}\;\mathrm{Width}\;w_{s}\;(\mathrm{mm})$	$\mathrm{Bar}\;\mathrm{Width}\;w_{b}\;(\mathrm{mm})$	V/A Ratio Samples (mm)	V/A Ratio Part (mm)
T0.5	0.5	40	3	2	0.24	1.18
T1.0	1.0	40	5	4	0.44	
T1.5	1.5	40	7	6	0.63	
T2.0	2.0	40	9	8	0.82	
T2.5	2.5	40	11	10	1.00	
T3.0	3.0	40	13	12	1.17	
T3.5	3.5	40	15	14	1.36	

**Table 2:** Overview of the concentration-dependent model parameters implemented. The expansion coefficients are valid for an O/V-ratio of 0.44 and are exemplarily selected.

Material Behaviour					
	Diffusivity	Swelling-Induced Deformation			
Water Concentration c	Diffusion Coefficient D	Young’s Modulus E	Expansion Coefficients for O/V = 0.44		
			a_11_	a_22_	a_33_
[ppm]	[m/s^2^]	[MPa]	[ppm^−1^]	[ppm^−1^]	[ppm^−1^]
0	2.58 $\times 10^{-9}$	1100	0	0	0
30,000	7.34 $\times 10^{-9}$	400	$1.06\times 10^{-7}$	0.86 $\times 10^{-7}$	2.78 $\times 10^{-7}$
65,000	2.42 $\times 10^{-8}$	350	1.17 $\times \;10^{-7}$	1.28 $\times 10^{-7}$	3.64 $\times 10^{-7}$
89,000	5.51 $\times 10^{-8}$	330	1.36 $\times \;10^{-7}$	1.81 $\times 10^{-7}$	3.93 $\times 10^{-7}$
